# Epstein-Barr virus, vitamin D and the immune response: connections with consequences for multiple sclerosis

**DOI:** 10.3389/fimmu.2024.1503808

**Published:** 2024-12-23

**Authors:** Atia Rasheed, Gulfaraz Khan

**Affiliations:** ^1^ Department of Medical Microbiology and Immunology, College of Medicine and Health Sciences, United Arab Emirates University, Al Ain, United Arab Emirates; ^2^ Zayed bin Sultan Center for Health Sciences, United Arab Emirates University, Al Ain, United Arab Emirates

**Keywords:** multiple sclerosis, EBV, vitamin D, immune system, molecular mimicry

## Abstract

Multiple sclerosis (MS) is an autoimmune disease of the central nervous system (CNS) with no definitive trigger. However, epidemiological studies indicate that environmental factors, such as infection with Epstein-Barr virus (EBV) and low vitamin D (Vit D) levels in genetically predisposed individuals, are important risk factors. One leading proposal is that EBV triggers MS via mechanisms such as molecular mimicry, where activated autoreactive B and T lymphocytes mistakenly target self-antigens. In line with other risk factors, low serum Vit D level, genetic polymorphism of Vit D receptor, and higher incidence of MS in countries in the northern hemisphere, suggest that Vit D also plays a role in MS pathology. Vitamin D, known for its neuroprotective and immunomodulatory effects, helps maintain a balance between pro-inflammatory and anti-inflammatory immune cells. Studies and ongoing clinical trials indicate that hypovitaminosis D is associated with an increased risk of MS, and Vit D supplement can help to reduce the disease severity. Moreover, hypovitaminosis D has also been associated with a dysregulated immune system and an increased risk of developing MS. This review explores how these three well-recognized risk factors - EBV infection, hypovitaminosis D, and dysregulated immune system - interact in the pathogenesis of MS. Understanding these interactions and their consequences could provide new insights into novel therapeutic approaches for treating this devastating disease.

## 1 Introduction

Multiple sclerosis (MS) is a chronic autoimmune-mediated disease with a complex etiology. It involves a dysregulated immune system with bouts of peripherally mediated inflammation and ongoing CNS-compartmentalized inflammation, leading to loss of myelin sheath and progressive worsening disability (1). The disease is believed to be initiated and driven by autoreactive lymphocytes that target myelin basic protein (MBP) (2). Normally, myelin sheath wraps around axons within the CNS, facilitating the rapid transmission of electric impulses. However, any severe damage to the myelin sheath can hinder the conduction of impulses and ultimately cause disability (2). Thus, MS is characterized by neurological symptoms manifesting at different times and locations. These symptoms tend to decrease as the underlying damage is repaired. Therefore, clinically, MS is classified into four subtypes based on its progression: relapsing-remitting (RRMS), primary progressive (PPMS), secondary progressive (SPMS), and progressive relapsing MS (PRMS) (3).

MS is a complex, multifactorial disease, and its exact cause remains unknown. However, genome-wide association studies (GWAS) have indicated many genetic variants contributing to MS susceptibility, including genes regulating the immune response (4). The strongest genetic association is due to variation in major histocompatibility complex (MHC) (i.e., HLA) class II alleles DRB1*0101, DRB1*0602, and DRB1*1501 (5). Apart from the genetic association, several environmental risk factors have also been implicated, such as lifestyle factors, smoking, exposure to organic solvents, heavy metals, infectious agents and hypovitaminosis D (6–8). Of the environmental factors, Epstein-Barr virus (EBV) and low vitamin D (Vit D) levels are considered strong risk factors for disease onset (8, 9). These risk factors could have an additive effect. For example, it has been shown that having EBV infection or HLA-DRB1*1501, or both, have a strong association in the development of MS (10). However, the role of other environmental factors in disease onset is limited (8).

## 2 EBV and MS

EBV is highly prevalent, infecting more than 90% of the global population. The infection is usually acquired early in childhood, generally with no pathological consequences. However, the virus is known to have oncogenic properties and is involved in the pathogenesis of several types of human malignancies (11, 12). EBV has also been implicated in the pathogenesis of MS (13). Epidemiological studies have shown that individuals who develop infectious mononucleosis (IM) following primary EBV infection have a 2-3 fold increased risk of developing MS later in life (14–16). Moreover, patients with MS have elevated levels of EBV-specific immune responses, which correlate with disease activity (17–20). By contrast, EBV-negative individuals have a significantly reduced risk of developing MS (21–23). Importantly, EBV-infected cells have been directly demonstrated in the brain of most cases of MS (24–26). More recently, in a rabbit model of EBV infection, it was shown that circulating EBV-infected cells can cross the blood-brain barrier (BBB) and induce inflammation and demyelination reminiscent of MS (27) To further galvanize the aetiological link between EBV and MS, a recent study involving more than 10 million active US military members, followed for over 20-years, found that 801 individuals developed MS. Of these, 35 cases were EBV seronegative and all but 1 case became infected with EBV before the onset of the disease. The authors concluded that EBV infection increases the risk of MS by 32 folds (28). These findings provide compelling evidence that EBV may serve as a trigger and potentially a driver for the development of MS (28, 29).

EBV (Human herpes virus 4) is a large dsDNA virus (11). Its genome is approximately 172kb long and encodes around 80 proteins and 46 functional small untranslated RNAs (miRNA). Some proteins are involved in viral genome replication and the generation of new viral particles during the lytic (productive) viral cycle, which is believed to occur primarily in B-cells (30). Herpesviruses, including EBV, are known for their ability to establish a latent phase of infection, where they persist within the host by expressing a limited number of genes that contribute to the virus’s ability to persist for life in its host (31).

EBV life cycle is characterized by lytic and latency programs (latency 0, I, II, and III) occurring within the infected B-cells. Throughout this life cycle, different viral proteins and miRNAs are produced, including six Epstein-Barr virus nuclear antigens (EBNA-1, 2, 3A, 3B, 3C, and LP), three latent membrane proteins (LMP-1, LMP-2A, and LMP-2B), two small non-coding RNAs (EBER-1 and 2) and dozens of miRNAs (31, 32). Of the EBV latent proteins, EBNA-1 is one of the most essential viral proteins expressed in all latency phases of the virus, except perhaps latency 0. It is necessary for viral DNA replication, episomal genome maintenance, expression of other latent proteins, immune evasion, and cell immortalization (33, 34). EBNA-1 is a multi-domain phosphoprotein having a DNA-binding and dimerization domain (DBD/DDD) within the C-terminal, involved in all EBNA-1 functions associated with binding to the origin of replication (oriP). On the other hand, its basic N-terminus consists of glycine-alanine domains (GAr) (aa 40-64 and aa 325-367), which are conserved across all EBV strains. The two GAr domains play critical roles in the ability of EBNA-1 to evade the immune system during the latent phase of viral infection (35). Therefore, EBNA-1 is highly antigenic, leading to the development of EBNA-1-specific autoreactive antibodies and cross-reactive T-cells in MS patients (36).

### 2.1 EBV-specific cross-reactive lymphocytes in MS

It has long been believed that MS is a T-cell-mediated disease (37). In several histopathological studies on MS lesions, T-cells were found to be much more abundant than B-cells (38). However, the importance of B-cells in MS pathology cannot be neglected; indeed, new therapeutic approaches, such as rituximab, target B-cells (39). Evidence for the involvement of B-cells in MS has been accumulating over the past 10-15 years, but their precise role in the evolution of the disease is still under discussion (39). Various studies have identified IgG oligoclonal bands in the CNS of MS patients that can recognize EBV antigens, particularly EBNA-1 (40, 41). Furthermore, the presence of ectopic B-cell follicles in the subarachnoid space and white matter lesions indicate the continuous activation of B and T-cells (41). Over 90% of MS patients have been reported to be positive for IgG oligoclonal bands, long-known as a diagnostic marker (42, 43). Additionally, patients with high levels of brain inflammation have been recognized by infiltrating B and T-cells into the meninges (44), perivascular cuffs, and brain parenchyma (45). These infiltrating cells lead to active lesions, demyelination, and progressive clinical disease course (46, 47). These findings indicate the involvement of both B and T-cells in MS pathogenesis.

### 2.2 T-cells in MS

**Figure 1:**
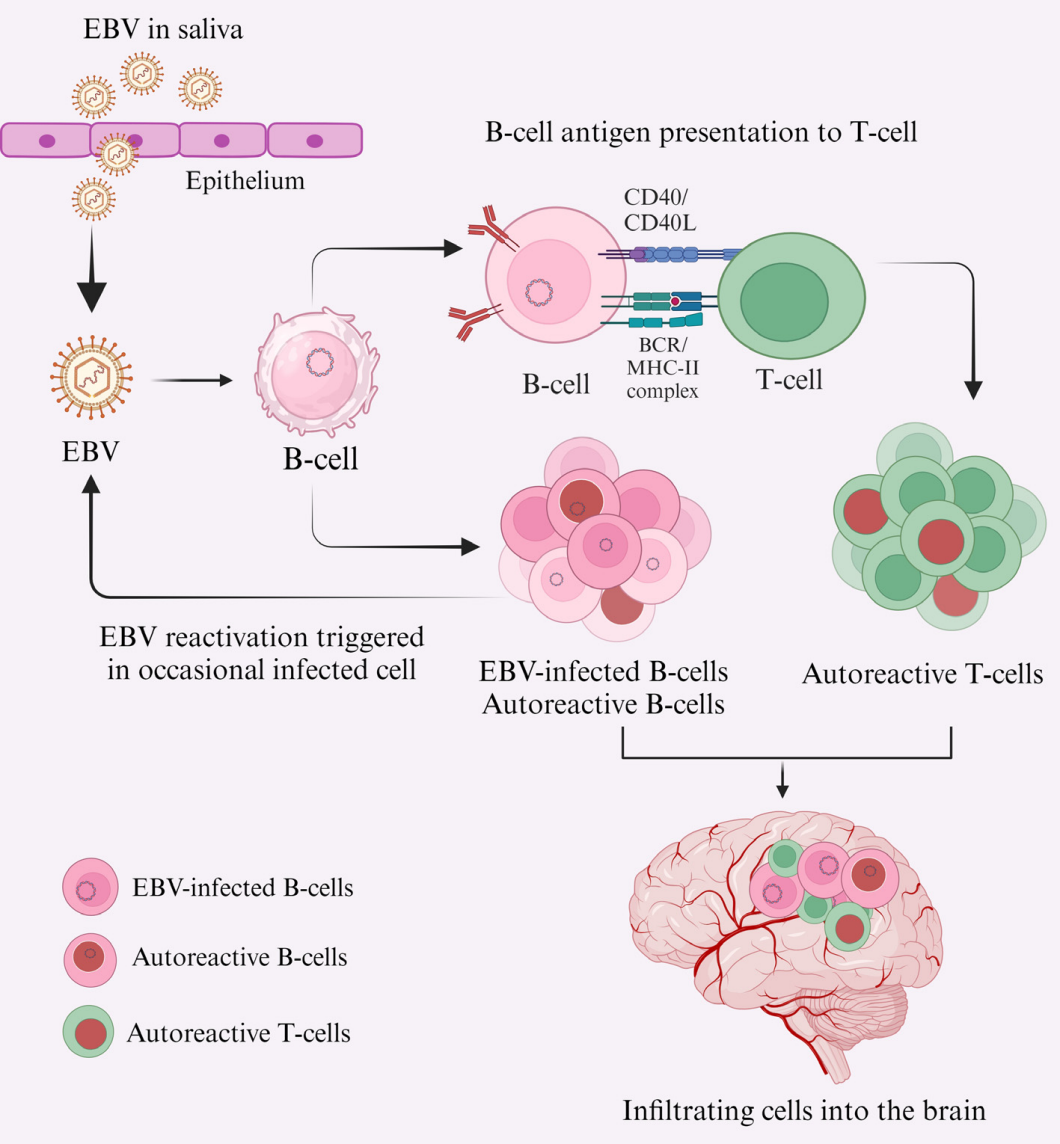
Primary EBV infection leads to CNS inflammation and MS lesions, potentially due to the infiltration of cross-reactive lymphocytes into the CNS. EBV is transmitted primarily via saliva and targets B-cells in the oral cavity, either directly or by oropharyngeal epithelial cells (61). Acute EBV infection can result in EBV-transformed B-cells, which may either transform into autoreactive B-cells (red nuclei) or act as antigen-presenting cells to T-cells (62), transforming them into autoreactive T-cells (red nuclei). These autoreactive lymphocytes cross the BBB and enter the CNS (63). Pathogenic and autoreactive B-cells secrete autoantibodies, particularly against EBNA-1, and autoreactive T-cells cross-react with self-antigens (e.g., MBP, GlialCAM, Anoctamin 2) (64, 65), contributing to MS pathogenesis.

MS patients have been reported to have self-reactive T-cells in their immune system; these cells can also exist in an inactive form in healthy individuals. However, their pathogenic effect is only realized when they become activated, which can occur due to various mechanisms (48). One hypothesis states that reactivation of EBV results in peripherally activated B and T lymphocytes crossing the BBB and entering the CNS (**Figure 1**), where they cross-react with self-antigens, resulting in local inflammation and tissue damage (49). How EBV triggers the activation and transmigration of these lymphocytes into the CNS and how infiltrating cells are involved in local MS pathology remains unknown.

Many theories have evolved about the involvement of EBV in MS pathogenesis. One hypothesis is that chronic EBV infection causes the generation of so-called “exhausted T-cells” due to continuous antigen presentation by B-cells (**Figure 1**), resulting in inappropriate T-cell mediated control of pathogenic B-cells (50). Consistent with this, in healthy individuals, CD8^+^ cytotoxic T-cells keep control over EBV infection by directly killing virus-infected B-cells (51), and CD4^+^ T-cells. However, the GAr domain of the EBNA-1 limits antigen presentation by reducing translation and proteasomal processing by MHC-I (52). Thus, it reduces the activation of CD8^+^ T-cells, typically involved in antigen recognition and presentation by MHC-I (52), thereby increasing viral load (53). However, in healthy individuals, CD4^+^ T-cells are primed to EBNA-1 (54). Moreover, EBNA-1-specific CD4^+^ T-cells in healthy individuals can recognize autologous EBV-transformed B-lymphoblastoid cell lines (B-LCL) and kill EBNA-1-expressing targets through CD95L/CD95-mediated pathway (55). Conversely, in MS patients, EBNA-1-specific CD4^+^ T-cells have been found to expand selectively and cross-recognize MS-associated MBP, leading to MS pathology (55). Further analysis revealed the presence of CD4^+^ T-cells against a specific sequence of EBNA-1 (55). Several studies have demonstrated a distinct immune response of cross-reactive T-cells in the CSF of MS patients, supporting the involvement of T-cells in MS pathology (56, 57).

Although most T-cells normally react to one specific antigen, studies in MS and long-term EBV carriers have shown that a high proportion of EBV-specific CD4^+^ and CD8^+^ T-cells are polyfunctional cells (PFCs) (58). PFCs originate from the central memory compartment with less functional avidities, but retain their antigen-specific proliferation capacity through IL-2 secretion. As these cells are less susceptible to activation-induced cell death, it has been assumed that they are essential in persistent antigen exposure and high viral load (58, 59). Interestingly, EBV-specific PFCs appear to have different subsets, including some CD107a^-^ CD4^+^ T-cells producing IFN-γ, MIP1-α, TNF-α, and IL-2. By contrast, CD107a^+^ CD8^+^ T-cells expressed only three of four cytokines (MIP1-α, TNF-α, and IL-2) (58, 60). CD107a is a degranulation marker, representing the potential cytotoxic function of the immune cells. Taken together, both types of T-cells produce MIP1-α to the level that it dominates the response of EBV-specific T-cells (56). The increased presence of these cells, combined with selective impairment of cytokines, indicates immune dysfunction driven by viral dominance and enhanced neuroinflammation. This, in turn, could be responsible for MS pathogenesis.

### 2.3 Molecular mimicry

How do autoreactive T-cells reach the CNS, and how do they trigger an autoimmune response? The concept of considering CNS as a secondary lymphoid organ led to studying the communication mechanisms between CNS and the immune system. Different studies have proposed that the CNS is capable of immune surveillance, in which autoreactive T-cells can induce autoimmunity. Several studies reported that CD4^+^ Th1/Th17 cells migrated from the peripheral to CNS through cytokine gradient and showed strong reactivity with the MS-associated myelin antigen in MS patients (55, 66). This evidence for autoreactive T-cells is the basis for molecular mimicry between viral epitope and MBP, supported by the mechanistic studies implicating EBNA-1 specific T-cell-mediated autoimmunity to myelin antigens, including proteolipid proteins (PLP), myelin oligodendrocytes glycoprotein (MOG) and MBP (49). Detailed analysis revealed the presence of autoreactive CD4^+^ T-cells with Th1 phenotype against EBNA-1 C-terminal spanning amino acids 400-641 (55). Moreover, autoantibodies against specific sequences of EBNA-1 have also been demonstrated in MS patients (67), indicating that EBNA-1 is a key viral protein triggering autoimmune responses in MS.

MS is associated with three HLA class II alleles belonging to haplotype HLA-DR2 (DR2) (10). DRB1*1501 and DRB5*0101 encode for the β-chain of DR2b and DR2a, respectively. MHC-II regulates the activation of CD4^+^ T-cells through the interaction of CD4^+^ TCR and MHC-II peptide complex, leading to signal transduction, activation, and differentiation of T-cells into different phenotypes (68). Analysis of antigen-specific TCR of a specific T-cell clone (Hy.2E11) from MS patients showed cross-reactivity with MBP and EBV antigens (69). Detailed analysis revealed that these two peptides are presented by different complexes: MBP complex is presented by DR2b and EBV peptides by DR2a (48). However, both complexes were found to have astounding similarities between them (69). Moreover, structural studies revealed the cross-reactivity of EBNA-1-specific T-cells to the N-terminal of MBP (residue 85-99) (55, 64), suggesting that the elevated level of these T-cells may target the MBP, which could provoke MS pathogenesis.

Another study on T-cell repertoire in MS patients, including identical twins, found more EBV-specific T-cell repertoire in MS patients than in healthy individuals (38). Interestingly, these T-cells were found to cross-react with the viral antigens (38), further strengthening the connection between EBV and MS.

### 2.4 B-cells in MS

Although cross-reactive T-cell-dominated inflammation is a characteristic of almost all types lesions, the presence of EBNA-1-specific autoantibodies has also been observed in the CNS of MS patients (67). Studies have revealed that these antibodies contribute to oligoclonal bands produced by clonal expansion of plasma cell-derived B-cells (42, 67). Several studies have reported the cross-reactivity of autoantibodies against the CNS autoantigens, including MBP, anoctamin 2, glial adhesion molecules, and αβ-crystallin, resulting in autoimmunity through molecular mimicry (64, 65). Like autoreactive T-cells, EBNA-1-specific antibodies are also raised against the C-terminal domain of EBNA-1 (385-420 residue). However, injection of EBNA-1-specific peptide (385-420 residue) into the MS mice model of EAE, results in CNS autoimmunity, further confirming the involvement of EBV-specific antibodies in MS pathology (67). Despite the overwhelming evidence for the presence of EBNA-1-specific B and T-cells in MS patients, the molecular mechanism for B-cell involvement is still poorly understood. One hypothesis is that, in addition to producing autoantibodies, B-cells may influence the activation and functioning of T-cells (70). As previously mentioned, targeting B-cells with rituximab, an anti-B-cell antibody, ameliorates symptoms of MS (71), further highlighting the critical role of B-cells in MS pathology.

### 2.5 Crosstalk between B and T-cells in MS

B-cells are multifunctional players in mediating both humoral and cellular immune responses. Additionally, B-cells are implicated in the formation of ectopic germinal center-like structures reported in the CNS of MS patients (44). Moreover, memory B-cells are also the prime target of EBV and take part in the development of T-cells in various ways, including activation of antigen-presenting cells (APC), expression of co-stimulatory molecules for effector T-cell function, and release of different cytokines (62). The range of cytokines from activated B-cells includes TNF, IL-6, and GM-CSF, which increase T-cell activation and contribute to the differentiation and proliferation of B-cells (39). EBV is a B-cell tropic virus, and a large pool of antigen-presenting B-cells is generated during virus-mediated activation (62). Some of these cells might persist for a long period, resulting in the emergence of exhausted or cross-reactive T-cells. Several *in vivo* studies reported the efficient stimulation of CD4^+^ T-cells by pathogenic B-cells, and they also supported their expansion during primary immune response (70, 72). Furthermore, EBV-transformed B-cells have been reported to efficiently activate and expand brain-homing CD4^+^ T-cells, particularly Th1 cells (62). These cells migrate into the CNS and cause local inflammation and tissue damage. *In vitro* study provided evidence that natalizumab, an MS treatment drug, blocks the migration of activated lymphocytes into the CNS (73). Interestingly, it was observed that the expansion of RAS guanyl-releasing protein 2 (RASGRP2), an autoantigen expressed in the brain and B-cells, impairs T-cells (62), suggesting that the crosstalk between pathogenic B and T-cells, can result in cross-reactive T-cells (**Figure 1**).

Although B-cells have been considered a cellular source of antibodies, it is clear that they can also regulate cellular and humoral immunity by producing cytokines that orchestrate the nature of the immune response. Like T-cells, B-cells can be polarized and make different cytokines, particularly IL-10, that have been implicated in controlling the immune response and are involved in CNS autoimmunity (74). Interestingly, B-regulatory cells (B-reg) are the primary producers of IL-10 and can maintain peripheral tolerance and suppress the development of autoimmune disease (74). This is further supported by *in vivo* studies in the EAE model (75). It was suggested that B-cells play an important role in controlling MS as B-cells depleted mice failed to recover after initial damage to the CNS (75). Several studies characterized the peripheral B-cells from MS patients and observed altered cytokine profile (76). Pathogenic B-cells in the blood released more pro-inflammatory cytokines, IL-6, GM-CSF, and decreased ability to produce IL-10 (76), suggesting an altered cellular functions in MS. Taken together, these findings indicate that B-cells are central players in MS pathogenesis because of their malfunctioning and hijacking by EBV. Moreover, the crosstalk between pathogenic B and T-cells could result in disease severity.

Despite 90% of the population being positive for EBV, only a very small proportion develop MS, indicating that factors beyond EBV are involved in the disease onset in genetically predisposed individuals. Another significant environmental risk is the low Vit D level, which is linked to an increased risk of MS. Conversely, maintaining a normal Vit D level, particularly in the early decades of life, can protect against the disease’s onset (77, 78). The ability of Vit D to modulate the immune system seems crucial in understanding how its deficiency may lead to incorrect programming of immune cells. Some malfunctioning cells then migrate into the CNS and target the MBP. Here, we describe the potential involvement of Vit D in MS pathogenesis by highlighting several potential mechanisms that could lead to EBV spread and autoimmunity.

## 3 Role of Vit D in MS

Vit D is increasingly considered as an important immune modulator (79). It functions as a steroid hormone, plays a crucial role in calcium and phosphate metabolism, immune homeostasis, and influences brain function during development and adulthood (77). Consequently, hypovitaminosis D has been associated with various diseases, including rheumatoid arthritis, type I diabetes, and autoimmune diseases (77, 80). There is an accumulating body of data that supports the association of circulating Vit D levels and MS with disease activity and progression. Different studies demonstrated a decrease of around 41% in MS risk with increased serum Vit D level (81). Several studies used Mendelian Randomization (MR) to measure the risk of MS associated with Vit D level (82–85). One study did an MR control trial on Vit D levels in MS in a large European population and reported that one standard deviation decrease in Vit D level in genetically predisposed individuals resulted in a 2-fold increased risk of developing MS (82). Additionally, one of the largest recent genome-wide association studies (GWAS) on serum Vit D level and MS included 401,406 participants, 24,091 controls, and 14,498 MS patients of European ancestry. The findings indicated an inverse correlation between MS and Vit D (86).

### 3.1 Vit D metabolism

**Figure 2:**
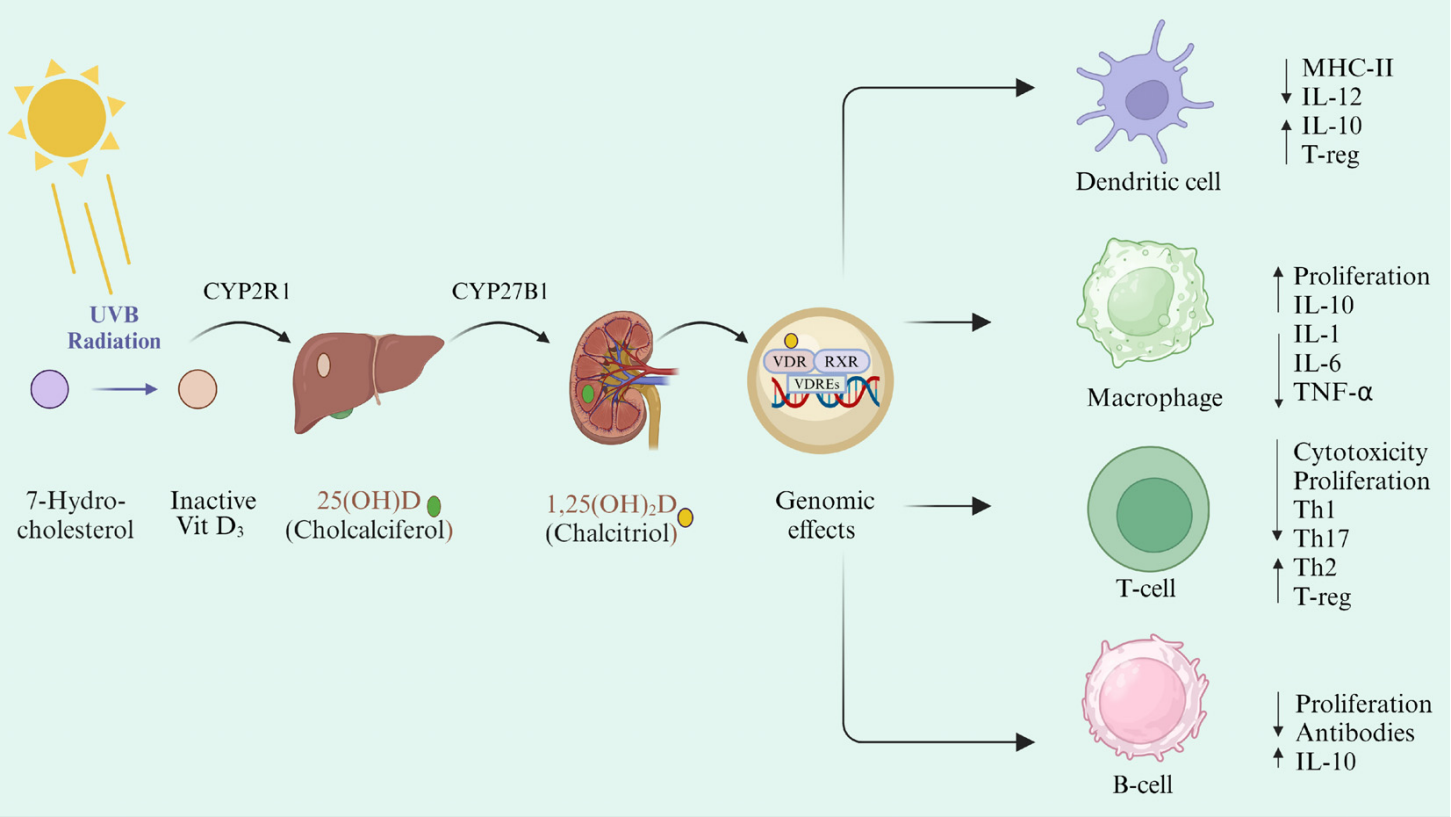
Vit D metabolism and its immunomodulatory action. Vit D can be either synthesized in the skin under the effect of UVB radiation or absorbed through dietary intake. Cholecalciferol is stored in the adipose tissues and hydroxylated in the liver into 25(OH)D by the CYP2R1 enzyme. 25(OH)D is then converted into 1,25(OH)_2_D by CYP27B1 in the kidney. Activated Vit D then circulates ubiquitously and binds to its transporter (VDBP). Calcitriol binds to cytoplasmic VDR, which makes the complex with RXR and is transported to the nucleus. Vit D/VDR/RXR complex binds to the VDRE on the DNA and regulates the expression of different genes (92). Vit D exerts a direct effect through binding with VDR on DC and T-cells and intervenes with their antigen-presenting function by decreasing MHC-II presentation on their surface. Vit D reduces the Th1 and Th17 differentiation and proliferation and shifts them toward tolerogenic immune response (93).

Vit D is mainly synthesized in the skin, with less than <5% coming from dietary intake. Upon exposure to ultraviolet B (UVB) radiation (sunlight), precursor Vit D (7-dehydrocholcholesterol) is transformed into Vit D_3_ (cholecalciferol) and then converted to biologically active form (calcitriol) in a two-step hydroxylation process (**Figure 2**). Firstly, it is converted to 25-hydroxyvitamin D (25(OH)D) by 25 hydroxylases (CYP27A1, CYP3A4, CYP2R1), mainly expressed in the liver. Later, 1-α-hydroxylase (CYP27B1), which is predominantly expressed in kidneys, converts 25(OH)D into biologically active form 1,25-dihydroxyvitamin D (1α,25(OH)_2_D) (calcitriol) (87, 88). Calcitriol forms the (1α,25(OH)_2_D)- Vit D receptor (VDR) complex and modulates the expression of around 500 genes (89, 90). Most of these genes are associated with Vit D metabolism and immunological processes (91), highlighting the involvement of Vit D in inflammatory diseases (**Figure 2**).

### 3.2 Genetic clues for Vit D involvement in MS susceptibility

Vit D receptor elements (VDREs) are regulated by Vit D and are present in the promotor region of more than 80% of MS-associated genes (94). Therefore, suboptimal Vit D level can result in the altered expression of MS susceptible genes, leading to MS predisposition (95). Vit D-binding protein (VDBP) plays an essential role in the regulation of Vit D availability to the target cells (96). The presence of VDBP in the CSF of MS patients further confirms the access of Vit D metabolites into the CNS and is proposed to play an important role in reducing the disease severity (97). However, the association between VDBP and MS is controversial, and their relationship still needs to be defined (95). Moreover, VDR and CYP27B1 were also observed in the healthy controls’ grey matter neurons and astrocytes, suggesting that these cells might be involved in Vit D regulation (98). Similarly, in an *in vivo* EAE experimental model, the increased expression of VDR and CYP27B1 in the CNS resulted in reduced inflammation and protection against severe EAE development (99), demonstrating the protective role of Vit D in autoimmunity. Interestingly, GWAS studies have found single nucleotide polymorphism (SNPs) in the CYP27B1 gene with a positive correlation to MS (96, 100). Likewise, a pilot study conducted in MS patients demonstrated an association between SNP in the CYP24A1 and CYP27A1 genes, Vit D levels, and risk of MS. A higher frequency of SNP in CYP24A1 gene and low Vit D levels were observed particularly in MS patients as compared to control (101). These findings further confirm a role for Vit D and its metabolites in MS susceptibility. However, the details of the mechanism remain unclear.

### 3.3 Role of Vit D in innate immunity in MS

The role of Vit D in the pathogenesis of MS could be more clearly understood by breaking it down into three distinct steps: (i) activation of autoreactive B and T-cells, (ii) disruption of BBB by autoreactive cells and infiltration into the CNS, (iii) effector function of infiltrated cells and progressive neurodegeneration (102). Vit D regulates the epigenetic programming of immune cells, promotes immunological tolerance in T-cells, and reduces the inflammatory response, all of which contribute to MS pathogenesis (77). Therefore, the appropriate level of Vit D should prevent the activation of autoreactive lymphocytes (77).

Vit D receptor is expressed intracellularly by various immune cells such as dendritic cells (DC), resting monocytes, macrophages, and natural killer (NK) cells (103, 104). Calcitriol influences the activity of these cells by downregulating the expression of MHC-II and promoting their tolerogenic activity (105, 106). It also inhibits the differentiation of monocytes into DC, hence downregulating IL-12 production. Additionally, Vit D regulates the expression of intracellular toll-like receptors (TLR) and reduces IL-6 production by downregulating the TLR9 expression (106–109). IL-6 and IL-12 are pro-inflammatory cytokines that help the body to control EBV infection (106). However, their role in MS pathogenesis is more complex and detrimental. Therefore, reducing IL-6 and IL-12 production could help to decrease MS pathogenesis related to EBV and molecular mimicry (**Figure 2**).

### 3.4 Effect of Vit D on B-cells in MS

It is known that Vit D modulates the adaptive immune system as it influences B and T-cell function (102). B and T lymphocytes express only little VDR in the resting stage and is upregulated upon activation. Additionally, CYP27B1 and CYP24A1 are also expressed by B-cells, CD4^+^, and CD8^+^ T-cells, suggesting local activation and regulation of Vit D by immune cells (110). Several studies have reported that Vit D influences the proliferation and differentiation of B-cells, decreasing the autoantibody production through B-cell apoptosis (93). *In vitro* exposure of activated naive B-cells (CD27^-^, CD19^+^, IgG^-^) with calcitriol results in the inhibition of B-cell differentiation into post-switch memory B-cells (CD19^+^, IgG^+^) and plasma cells (CD38^+^, CD27^+^), leading to diminished antibody production (111). These findings demonstrate that Vit D may play a role in maintaining B-cell homeostasis; therefore, optimal Vit D level could be beneficial in diminishing MS pathology. Some recent studies have demonstrated a significant decrease in anti-EBNA-1 IgG titer in MS patients supplemented with calcitriol, with no effect on other viruses or EBV antigens (112).

It has been reported that *in vitro* Vit D promotes the production of IL-10-producing B-cells/B-reg and inhibits the co-stimulation of T-cells (113). Unfortunately, these studies are not supported by *in vivo* studies, possibly due to *in vivo* interaction of Vit D and its metabolites interfering with Vit D pathway and/or the interaction between EBV and B-cell in MS (113). In some clinical studies, no significant correlation was observed between Vit D and B-cell differentiation and antibody production (114, 115). Likewise, no correlation was found between serum Vit D level and B-reg in a cohort of RRMS patients and healthy controls (116). However, this does not diminish the effect of Vit D on the B-cell subset, including B-reg. It is possible that Vit D may affect the function of these cells through different mechanisms. Additional research is needed to understand their correlation, which might have clinical implications, including ongoing Vit D trials as an add-on MS therapy.

### 3.5 Effect of Vit D on T-cells in MS

**Figure 3:**
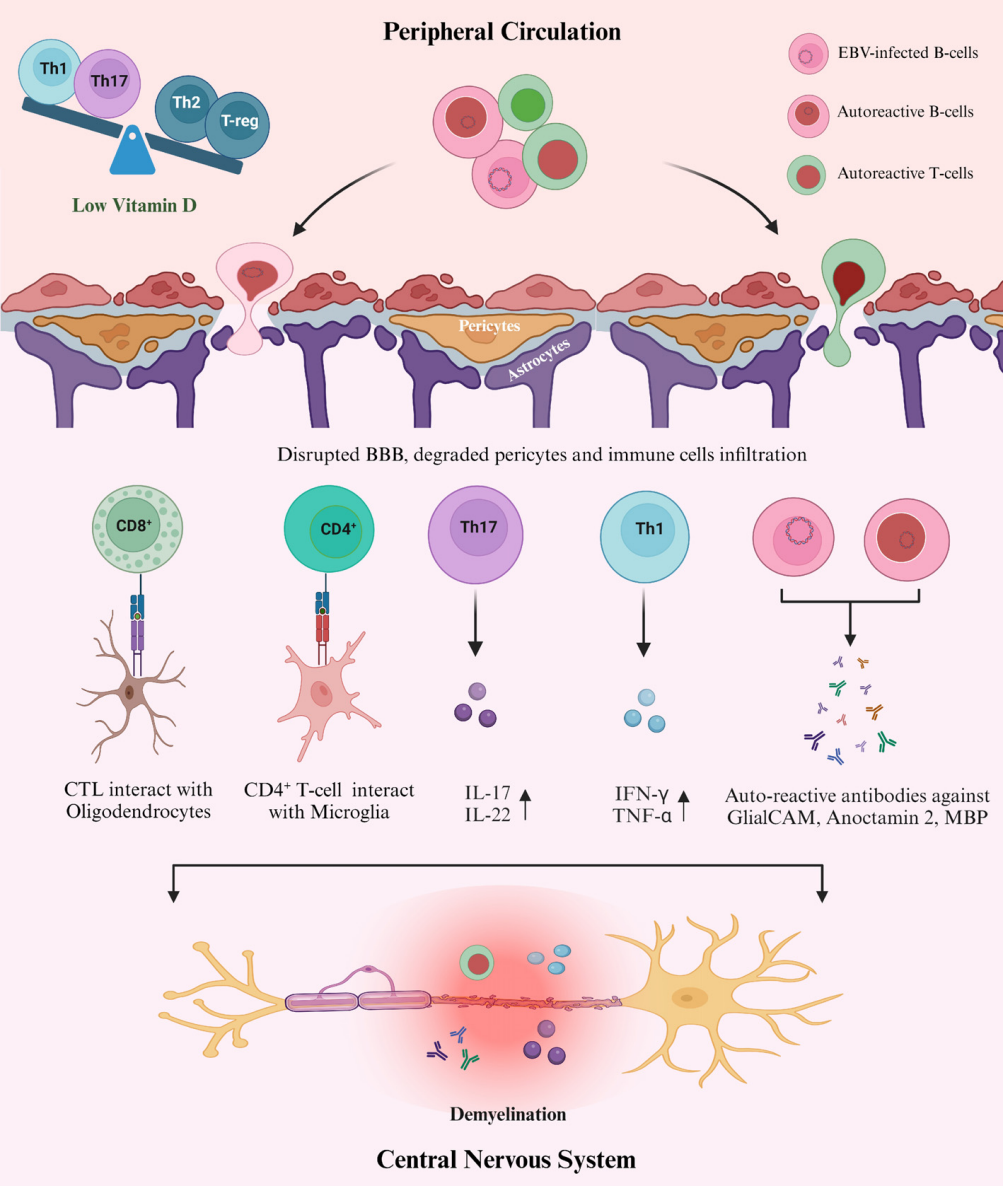
Proposed mechanism of how EBV, Vit D, and Aberrant immune response interact in the pathogenesis of MS. Sufficient levels of Vit D modulate the immune system in several ways, such as maintaining the balance between Th subsets and sustaining BBB integrity. However, hypovitaminosis D is likely to be associated with an imbalance between anti-inflammatory and pro-inflammatory immune cells in MS, which could also benefit the EBV spread and the production of autoreactive lymphocytes (119). Furthermore, the exact mechanism for the involvement of Vit D in maintaining CNS homeostasis is unknown. Still, the data suggests a direct relationship between low Vit D and an increased rate of infiltrating cells into the CNS through loss of pericytes and disrupted BBB (129). Infiltration of malfunctioning immune cells into the CNS results in cross-reaction with the self-antigens in the CNS either directly or by secreting autoantibodies (anti-EBNA-1 antibodies) or pro-inflammatory cytokines (47, 65), leading to MS pathology.

As part of adaptive immunity, Vit D directly affects T lymphocytes by inhibiting their proliferation at the G1_a_ to G1_b_ cell cycle phase (117). It also targets Th cells to regulate the balance between Th1, Th2, and Th17 cells (118) (**Figure 3**). This immunomodulatory activity of Vit D acts against the pathogen through several mechanisms, including the downregulation of pro-inflammatory cytokines that differentiate T-cells in Th1 and Th17 subsets (119). Vit D also promotes the differentiation of Th2 cells, producing anti-inflammatory cytokines (IL-3, IL-4, IL-5, IL-10) (119), which contribute beneficially to MS pathogenesis. However, the role of Vit D in controlling EBV has not been investigated yet. The immune response to EBV involves antigen presentation, activation and expansion of T-cells, which are modulated by Vit D_3_ in both *in vivo* and *in vitro* (120). The adaptive effect of Vit D also includes influence on regulatory T-cells (T-reg). This is further evident in a clinical study that suggested a positive association between serum Vit D level and T-reg in MS patients (121). This further supports the hypothesis that Vit D is an important factor in regulating the balance between Th cells in MS. Moreover, low Vit D level is associated with a low level of CD4^+^/CD8^+^ T-cells, which directly kill the virus-infected cells such as EBV-infected B-cells (122). B-cells are the prime target of EBV, and the virus persists in host memory B-cells to maintain different latencies. Thus, insufficient Vit D level may impair the ability to control EBV infection, hindering CD8^+^ T-cell production (123). Overall, an adequate level of Vit D plays a significant role in regulating the immune system by maintaining the balance between pro-inflammatory and anti-inflammatory cells, which is essential for controlling both EBV and MS (**Figure 3**).

### 3.6 Vit D and BBB integrity

Peripheral immune regulation by Vit D protects against CNS inflammation by regulating the activation of microglia and astrocytes in the brain parenchyma and maintaining BBB integrity (92). Within the BBB, endothelial cells are joined together through tight junctions. These cells are surrounded by pericytes and astrocytes, responsible for controlling the cellular exchange between blood and CNS (124). Pericytes are mural cells involved in sustaining BBB integrity and remyelination of the CNS lesions in MS. A recent study reported a direct relation between the loss of pericytes and the rate of infiltrating immune cells into the CNS in the EAE mouse model of MS (125). It was demonstrated that pericytes directly interact with T-cells and may act as non-professional antigen-presenting cells. This affects the activation and proliferation of T-cells and suggests that pericytes shape the functions of T-cells during their transmigration into the CNS after antigen-specific interaction (125) (**Figure 3**). Furthermore, different studies conducted in MS patients illustrate the presence of disrupted BBB, damaged pericytes, and increased rate of infiltrating immune cells into the CNS (126, 127). Further analysis revealed that Vit D is involved in maintaining the integrity of BBB in different ways, by reducing the rate of apoptosis of endothelial cells and inhibiting the loss of tight junctions to increase the survival rate of these cells in MS (128, 129). Furthermore, substantial evidence has been presented that demonstrates the effective role of Vit D in maintaining BBB integrity by using animal models (78, 129, 130). Taken together, studies have indicated that Vit D plays a crucial role in maintaining immune cell trafficking into the CNS and is also responsible for maintaining BBB integrity. However, the exact molecular mechanisms involved remain to be elucidated.

### 3.7 Outstanding questions

There are several outstanding questions pertaining to the interaction of EBV, Vit D and the immune system that need to be addressed. Studies have demonstrated that Vit D supplementation in MS patients can improve disease symptomology (131, 132). The Endocrine Society recommends that for general health, adults aged 19-50 years can take Vit D dose of 1500-2000 IU/day (87). However, it is unclear what dose is most effective in MS patients. A recent *in vivo* study using the EAE model of MS reported that high Vit D levels can exacerbate the disease (133). Moreover, despite extensive research on the immunomodulatory role of Vit D, its interaction with EBV is still not well known. *In vivo* studies aimed at addressing the effect of Vit D on EBV, the immune response against the virus, and its associated neuroinflammation could shed light on the interactions of these risk factors in MS pathogenesis. Furthermore, it is unclear if Vit D and EBV are the initiators or drivers of disease pathogenesis. The recent establishment of a rabbit model of EBV infection may help to address some of these central questions (27).

## 4 Conclusion

There is substantial evidence that EBV, low Vit D, and aberrant immune response are key players in the pathogenesis of MS. The details of how these three risk factors interact to trigger and drive MS remains unknown. We propose a model (**Figure 3**) in which persistent EBV infection results in malfunctioning B and T-cells that cross the BBB and enter the CNS. These cross-reactive infiltrating cells target self-antigens such as MBP, anoctamin 2, GlialCAM, and αβ-crystallin, resulting in MS pathogenesis. Low Vit D levels perturb the immune homeostasis, favoring the spread of EBV-infected cells and thereby further exacerbating the aberrant immune response. Delineating the interactions between these three risk factors and their consequences to the pathogenesis of MS, will help to shed light on strategies for potential interventions to prevent or at least reduce the burden of MS.
